# Integrated multi-omics analysis of genomics, epigenomics, and transcriptomics in ovarian carcinoma

**DOI:** 10.18632/aging.102047

**Published:** 2019-06-29

**Authors:** Mingjun Zheng, Yuexin Hu, Rui Gou, Jing Wang, Xin Nie, Xiao Li, Qing Liu, Juanjuan Liu, Bei Lin

**Affiliations:** 1 Department of Obstetrics and Gynecology, Shengjing Hospital Affiliated to China Medical University, Shenyang, Liaoning 110004, China; 2 Key Laboratory of Maternal-Fetal Medicine of Liaoning Province, Key Laboratory of Obstetrics and Gynecology of Higher Education of Liaoning Province, Liaoning, China

**Keywords:** multi-omics, ovarian carcinoma, molecular subtype, biomolecular markers, UBB, IL18BP

## Abstract

In this study, we identified prognostic biomarkers in ovarian carcinoma by integrating multi-omics DNA copy number variation (CNV) and methylation variation (MET) data. CNV, MET, and messenger RNA (mRNA) expression were examined in 351 ovarian carcinoma patients. Genes for which expression was correlated with DNA copy-number or DNA methylation were identified; three ovarian carcinoma gene subtypes were defined based on these correlations. Overall survival and B cell scores were lower, while the macrophage cell score was higher, in the DNA imprinting centre 1 (iC1) subtype than in the iC2 and iC3 subtypes. Comparison of CNV, MET, and mRNA expression among the subtypes identified two genes, ubiquitin B (UBB) and interleukin 18 binding protein (IL18BP), that were associated with prognosis. Mutation spectrum results based on subtype indicated that UBB and IL18BP expression may be influenced by mutation loci. Mutation levels were higher in iC1 samples than in iC2 or iC3 samples, indicating that the iC1 subtype is associated with disease progression. This integrated multi-omics analysis of genomics, epigenomics, and transcriptomics provides new insight into the molecular mechanisms of ovarian carcinoma and may help identify biomolecular markers for early disease diagnosis.

## Introduction

Ovarian carcinoma is the third most common type of gynecological malignancy [1]. Due to late detection, 70% of patients present with advanced cancer with distant metastasis upon diagnosis, and ovarian carcinoma is the leading cause of death among malignant gynecological tumors [2,3]. Both early detection biomarkers for ovarian carcinoma and effective therapies for recurrent cases are lacking [4]. It is therefore of clinical importance to identify effective tumor markers and investigate their role in the occurrence and development of ovarian carcinoma to aid in early diagnosis, prevention, and control of ovarian carcinoma [5].

A recent large-scale, multi-omics analysis of various cancers provides many new insights regarding dysregulation of gene levels in cancer [6]. Additionally, genomic variations caused by copy number variations (CNVs) or single nucleotide variations (SNVs) contribute to tumor occurrence and progression [7]. Furthermore, epigenetic regulation via DNA methylation (MET) in cancer genomics plays a key role in variation in disease characteristics [8]. Omics analyses in specific cancers, including hepatocellular carcinoma, demonstrate that wide variety of genomic and epigenomic dysregulations can affect cancer occurrence and progression [9]. CNV is a crucial regulator of genomic and epigenomic dysregulation that contribute to tumor progression and transcriptional dysregulation. These public, large-scale, multi-omics data sets make it possible to conduct an integrated multi-omics analysis of the impacts of genomics, epigenomics, and transcriptomics on tumor occurrence and progression in ovarian carcinoma [10].

CNV plays an important role in individual genetic variation and in human genetic diversity, and mutation rates are higher in genes that display CNV. CNV can alter gene expression by regulating mRNA levels and by influencing transcriptional regulation, and many CNVs are closely related to various diseases [11,12]. Walker *et al*. conducted a genome-wide association analysis of CNVs and risk of ovarian carcinoma in a cohort of 2,500 patients with the breast cancer type 1 (BRCA1) pathogenic variant. They found that the absence of CNV at the cyp2a7 locus (19q13.2) was correlated with a reduced risk of ovarian carcinoma (RR=0.50, p=0.007) [13]. A study of 330 families with increased rates of ovarian carcinoma identified three new pathogenic CNVs. Of these CNVs, BRCA1 (exon 4-13 absence, exon 12-18 absence) and ATM (exon 57-63 absence) were potentially associated with susceptibility to ovarian carcinoma [14]. Further investigations focused on CNVs will improve understanding of the molecular mechanisms of complex diseases and help identify susceptible genes [15,16]. However, many other genetic factors are important in cancer, and additional analyses are therefore required. Epigenetic inheritance refers to hereditary genetic changes that occur without any changes in the DNA sequence, including histone modification, DNA MET, RNA editing, and gene silencing. The occurrence and progression of ovarian cancer involve several functional pathways, including DNA repair, cell apoptosis, and cell cycle regulation, and are affected by changes in protooncogenes tumor suppressor genes. Studies have suggested that epigenetic changes in these pathways contribute to the development of ovarian cancer, and DNA MET may serve as a helpful biomarker for early diagnosis [17,18].

Because CNVs and DNA MET are involved in many types of cancer, it is important to investigate their effects on cancer progression [19]. In this study, CNVs, DNA MET, and mRNA expression were measured in 351ovarian carcinoma samples with clinical information. The relationships between mRNA expression and both CNV and MET were examined separately to identify a CNVcor gene set and a METcor gene set. The CNVcor and METcor genes were used together to identify for molecular subtypes in ovarian carcinoma, and specific targets or biomarkers that drove these subtype classifications were examined.

## RESULTS

### Comparison of CNVcor and METcor gene sets

**Figure 1:**
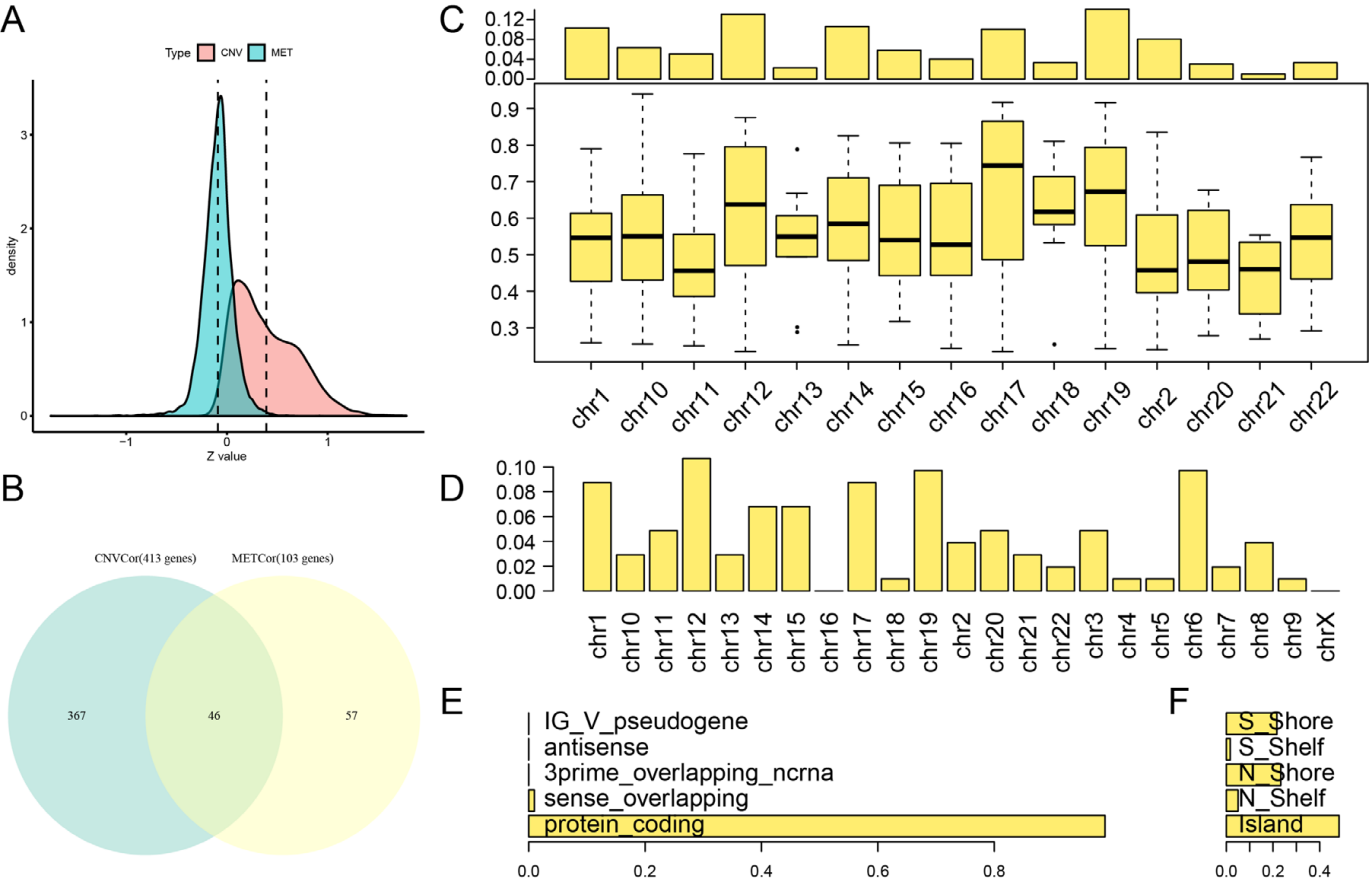
**Identification of DNA copy number-correlated (CNVcor) and DNA methylation-correlated (METcor) genes in ovarian carcinoma.** (**A**) Correlation z-value distributions for CNVcor genes and METcor genes. Density distribution is shown on the y-axis; the dotted line represents the median CNV and MET correlation coefficient values. (**B**) Venn diagram of overlapping CNVcor genes and METcor genes. The green area represents the 413 CNVcor genes. The yellow area represents the 103 METcor genes. The overlapping region contains 46 genes. (**C**) Box plot of CNVcor gene chromosomal distribution (upper figure) and correlations (lower figure). In the top image, the proportion of CNVcor genes on each chromosome (adding to a total of 1) is shown on the y-axis; in the bottom image, the correlation coefficient of CNVcor genes on each chromosome is shown on the y-axis. (**D**) Box plot of METcor gene chromosomal distribution. The proportion of METcor genes on each chromosome is shown on the y-axis. (**E**) METcor gene types. The proportion of METcor genes by type is shown on the x-axis. F: Distribution of MET loci. The proportion of types of methylated loci is shown on the x-axis.

A total of 3,990 CNVcor genes and 9,651 METcor genes were identified at a significance level of p < 0.01. In the z-value distribution, CNVcor gene correlations were significantly shifted to the right, while METcor gene correlations were significantly shifted to the left (Figure 1A) (D'Agostino test: p < 1e-5). These results indicate a positive correlation between CNVcor genes and gene expression and a negative correlation between METcor genes and gene expression. Due to the large number of genes in these two sets, only those genes significantly related to prognosis in each set (p < 0.05, 413 CNVcor genes and 103 METcor genes) were included in subsequent analyses. There was no significant overlap between the two gene sets (Figure 1B), indicating a possible lack of interaction between CNVcor and METcor genes. Further analysis of the genomic distribution of CNVcor and METcor genes revealed that CNVcor is more inclined to appear on chr14, chr11, chr12 and chr1 chromosomes (FDR< 0.05), while METcor is more inclined to appear on chr12 and chr15, chr14 and chr16 (p < 0.05) (Figures 1C, 1D,  S1, Table 1) and consist primarily of protein-coding genes (Figure 1E). The MET loci were primarily located in the S-shore, N-shore, and island regions (Figure 1F).

**Table 1:** Fisher significance test for the distribution of CNVCor and METCor genes on chromosomes.

CNVCor	gCount	FisherP	FDR(BH)
chr14	42	0.00001	0.00008
chr11	20	0.00059	0.00825
chr12	52	0.00074	0.00966
chr1	41	0.00165	0.01981
chr16	16	0.03923	0.43156
chr19	56	0.09318	0.93179
chr18	13	0.11366	1.00000
chr15	23	0.27520	1.00000
chr2	32	0.30108	1.00000
chr20	12	0.30654	1.00000
chr21	4	0.33219	1.00000
chr17	40	0.53937	1.00000
chr10	25	0.58392	1.00000
chr13	9	1.00000	1.00000
chr22	13	1.00000	1.00000
NA	15	NA	NA
METCor			
chr12	11	0.023056	0.530277
chr16	0	0.02405	0.530277
chr15	7	0.036821	0.773233
chr14	7	0.044257	0.88513
chr6	10	0.070549	1
chr21	3	0.123508	1
chr5	1	0.138885	1
chr4	1	0.189819	1
chr9	1	0.192522	1
chr20	5	0.210286	1
chr17	9	0.216074	1
chr13	3	0.237298	1
chr7	2	0.334773	1
chr19	10	0.344671	1
chr2	4	0.415739	1
chr11	5	0.687229	1
chr1	9	0.745382	1
chr8	4	0.78068	1
chr10	3	1	1
chr18	1	1	1
chr22	2	1	1
chr3	5	1	1
chrX	0	1	1

### Molecular subtypes were identified based on CNVcor and METcor genes

**Figure 2:**
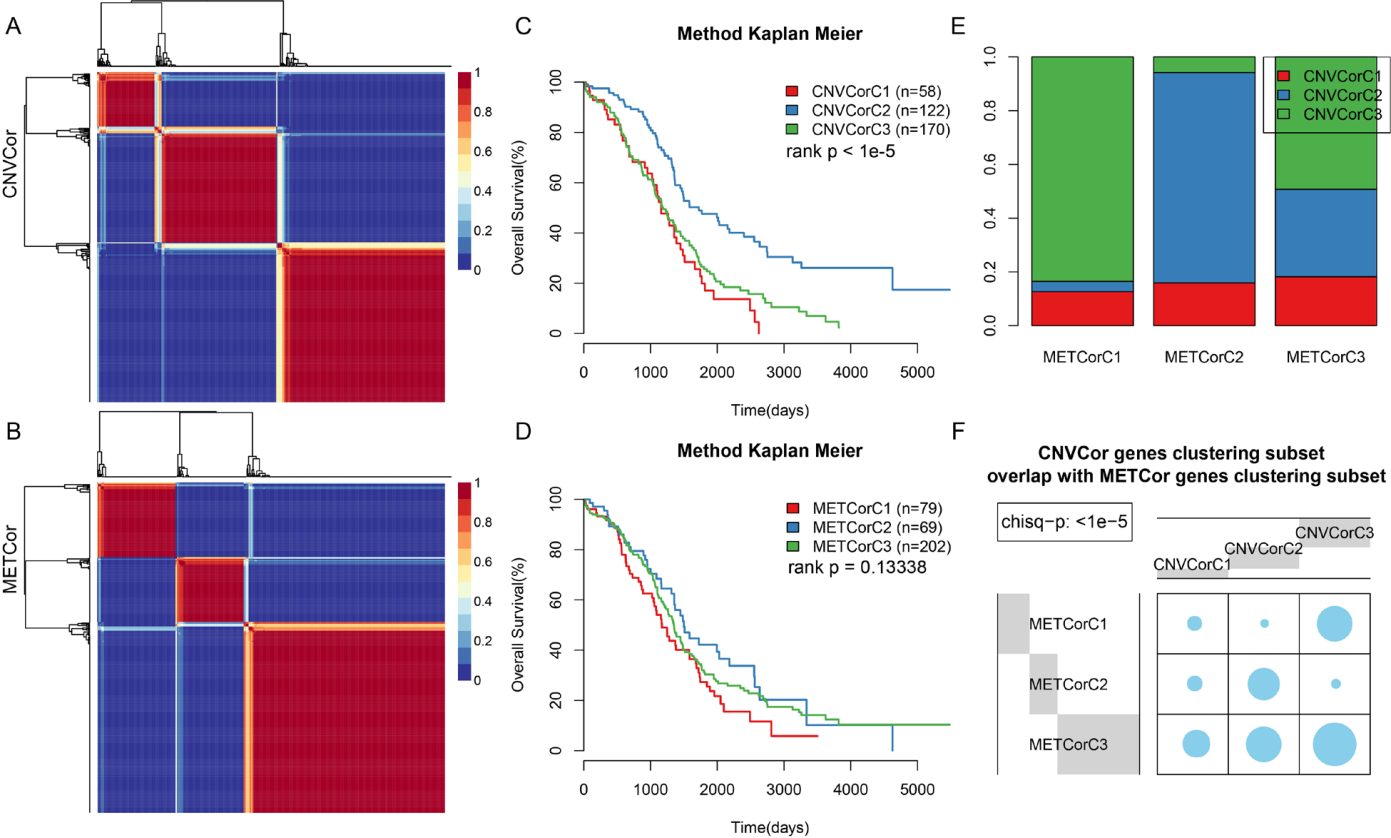
**Identification of ovarian carcinoma molecular subtypes based on CNVcor and METcor genes.** (**A**) NMF clustering results for CNVcor genes. (**B**) NMF clustering results for METcor genes. (**C**) KM survival curve for CNVcor gene clustering subsets. Survival time is shown on the x-axis, and survival rate determined by log rank P test is shown on the y-axis. (**D**) KM survival curve for METcor gene clustering subsets. Survival time is shown on the x-axis and survival rate determined by log rank P test is shown on the y-axis. (**E**) Overlap between CNVcor and METcor gene clustering subsets. F: Overlap test for CNVcor and METcor gene clustering subsets. Blue circles represent the proportion of overlapping samples between two the clusters; significance was determined using the Kolmogorov-Smirnov test.

The CNVcor and METcor gene sets were clustered using non-negative matrix factorization (NMF). The standard “brunet” was selected by NMF and then subjected to 50 iterations. The clustering number k was set at 2-10. The average contour width of the sharing membrane matrix was determined by NMF in the R package, and the minimal member number for each subtype was set at 10. The optimal clustering number was determined using cophenetic, dispersion, and silhouette. The optimal clustering number was two for CNVcor genes (Figure 2A, Figure S2) and three for METcor genes (Figure 2B, Figure S3). Because no significant differences were observed when the CNVcor genes were clustered into three subtypes rather than two, and because the optimal clustering number for METcor genes was three, the optimal clustering number for CNVcor genes was also set at three for subsequent analyses. There were significant differences in OS for both the CNVcor and METcor genes in the three subtypes (Figure 2C, 2D, Figure S4, Table S4). Considerable overlap was observed between the CNVcor and METcor genes clustered into subtype three (Figure 2E and 2F, p < 0.05).

### CNV, MET, and EXP data were integrated to divide samples into four categories

**Figure 3:**
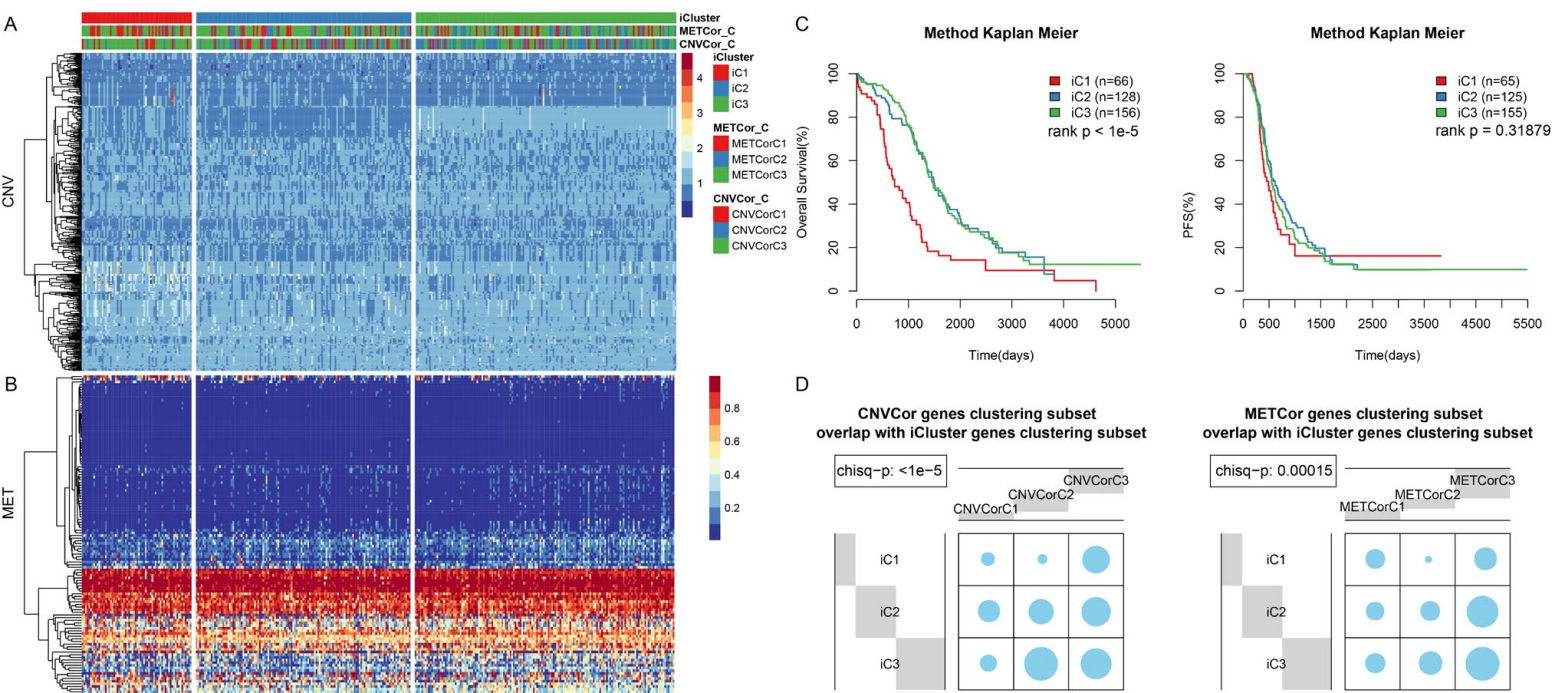
**OV sample molecular typing results in multiple datasets.** (**A**) Expression heatmap for CNVcor gene subsets identified by iCluster. (**B**) Expression heatmap for METcor gene subsets identified by iCluster. (**C**) OS and PFS curves for subtypes identified by iCluster. (**D**) Overlap between METcor and CNVcor gene subsets with iCluster gene subsets. Blue circles represent the proportion of overlapping samples between two clusters; significance was determined using the Kolmogorov-Smirnov test.

Because METcor genes were clustered into three subtypes, K= 2-3 was chosen when calculating lambda values in the multi-omics clustering analysis. The final lambda values obtained at K=2 and 3 (3 and 4 clustering subtypes) were 0.004950495, 0.391089109, and 0.836633663, respectively. A considerable difference in sample size distribution among the three clustering subtypes was noted at K=2. Consequently, K=3 and 4 clustering subtypes with sample sizes of 1, 65, 128, and 156 were selected. Because the minimal clustering number cannot be less than 10, the subtypes containing one and 65 samples were merged into a single subtype. The final three resulting subtypes were iC1 (66 samples), iC2 (128 samples), and iC3 (156 samples). The clustering results for the three datasets are presented in Figure 3A and 3B, and clustering information is shown for each sample in Table S5.

OS (Figure 3C, p < 0.001), but not PFS (Figure 3C, p>0.05), differed significantly among the 3 subtypes. In addition, there was considerable overlap between the iCluster gene clustering results and the CNVcor and METcor gene clustering results (Figure 3C, 3D, p < 0.001).

### Correlations between CNV and MET variation

**Figure 4:**
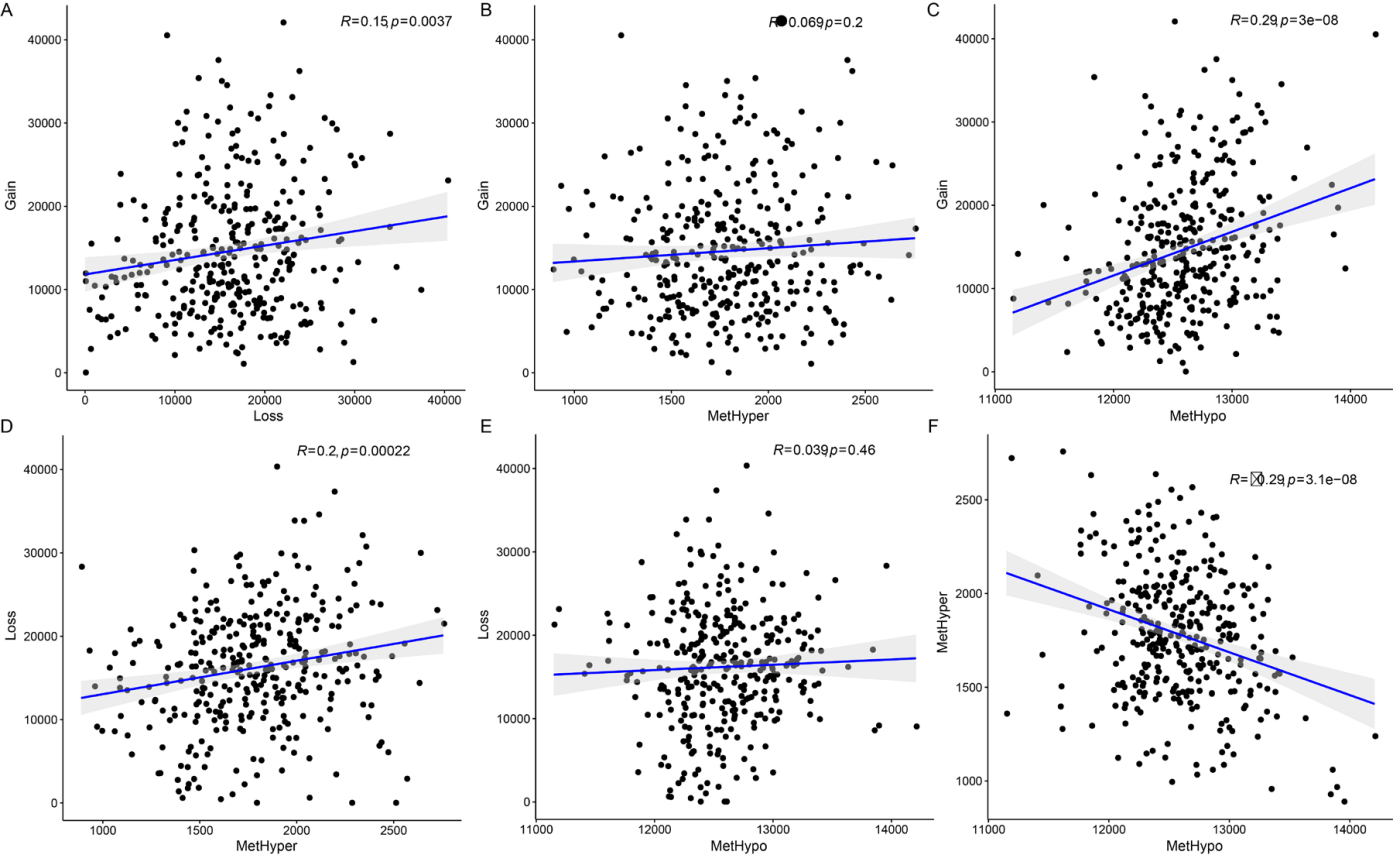
**Associations between aberrations in DNA copy number and DNA methylation in ovarian carcinoma.** (**A**) Frequency distribution of CNV Gain and CNV Loss. (**B**) Frequency distribution of CNV Gain and MetHyper. (**C**) Frequency distribution of CNV Gain and MetHypo. (**D**) Frequency distribution of CNV Loss and MetHyper. (**E**) Frequency distribution of CNV Loss and MetHypo. (**F**) Frequency distribution of MetHyper and MetHypo. Larger correlation coefficients (R values) indicate stronger correlations; the log rank P test was used.

Next, we examined the relationship between CNV and MET variation. For CNVs, CNV Gain was defined by a β>0.3 and CNV Loss by a β<-0.3; for MET, hypermethylation (MetHyper) was defined by a β>0.8 and hypomethylation (MetHypo) by a β<0.2. The number of genes showing CNV Gain, CNV Loss, MetHyper, and MetHypo was counted separately for each sample. CNV Gain and CNV Loss were positively correlated (R=0.15, p=0.0037) (Figure 4A), but there was no correlation between CNV Gain and CNV MetHyper (Figure 4B). There was also a strong positive correlation between CNV Gain and MetHypo (R=0.29, p=3e-08) (Figure 4C); CNV Loss and MetHyper were positively correlated as well (R=0.2, p=0.00022) (Figure 4D). There was no significant correlation between CNV Loss and MetHypo (Figure 4E). Finally, MetHyper and MetHypo were negatively correlated (R=-0.29, p=3.1e-08) (Figure 4F, Table S6).

### Immune scores differ among Ovarian Cancer (OV) gene subtypes

**Figure 5:**
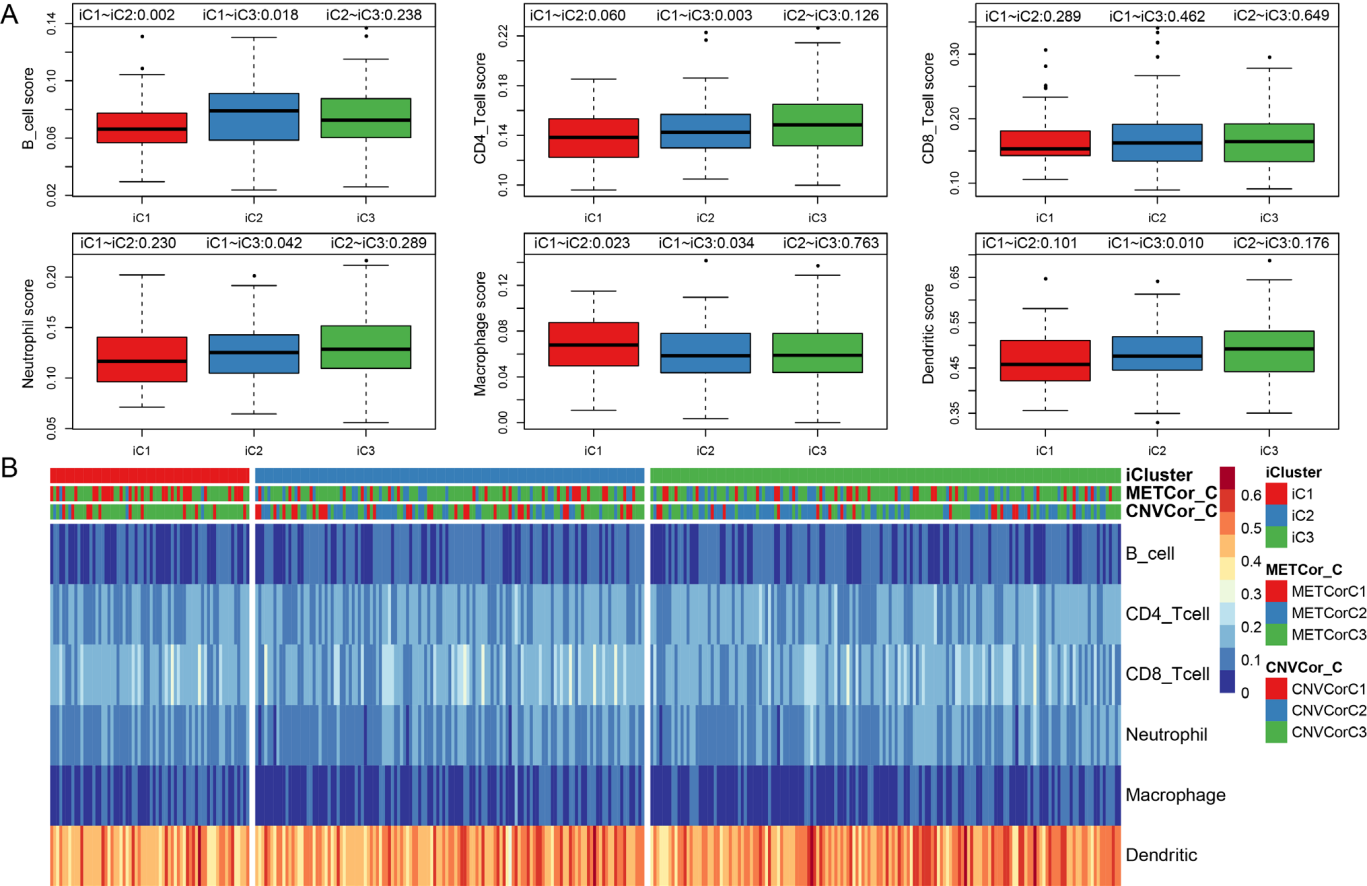
**Infiltration of iC molecular subtypes in different immune cells.** (**A**) Comparison of scores for six types of immune cells among the three iCluster gene subtypes. Immune cell scores are shown on the y-axis, and different molecular subtypes are shown on the x-axis. The Kruskal-Wallis rank test was used to identify significant differences in immune cell scores between the iC molecular subtypes. (**B**) Scores for six types of immune cells in all samples. iCluster indicates multi-omics molecular subtypes, METcor_C indicates METcor gene molecular subtypes, and CNVcor_C indicates CNVcor gene molecular subtypes.

All OV genes included in this study were clustered into three subtypes based on available multi-omics data. No significant differences in clinical characteristics (gender and age) were found among the three subtypes (Table 2). Immune scores were determined for samples grouped by subtype using tumor immune estimation resource (TIMER) tool. Compared to the other two subtypes, B cell scores were lower, while macrophage cell scores were higher, in iC1 subtype samples, which were also associated with the poorest prognoses (Figure 5A, p < 0.01). The immune scores of iC1 subtypes in CD4 T cells, neutrophils and dendritic cells are significantly lower than that of iC3 (P<0.05). We speculate that these molecular subtypes have different cellular immune functions and affect the survival and prognosis of patients to a certain extent.

**Table 2:** Comparison of clinical features of OV gene subtypes.

Event	Total	iC1	iC2	iC3
Alive	136	13	55	68
Dead	212	51	73	88
X	1	0	0	0
NewEvent				
0	119	26	43	50
1	230	38	86	106
Grade				
G1_G2	43	6	18	19
G3_G4	300	57	108	135
GX	6	1	3	2
Stage				
II	20	4	6	10
III	274	55	103	116
IV	53	5	20	28
X	2	0	0	2
Age				
0~50	75	3	27	45
50~60	109	16	38	55
60~70	84	17	29	38
70~80	66	22	30	14
80~100	15	6	5	4

Scores for the six immune cell types examined in the three sample subtypes are shown in Figure 5B; the associated data are shown in Table S7.

### Molecular characteristics of OV gene subtypes

**Figure 6:**
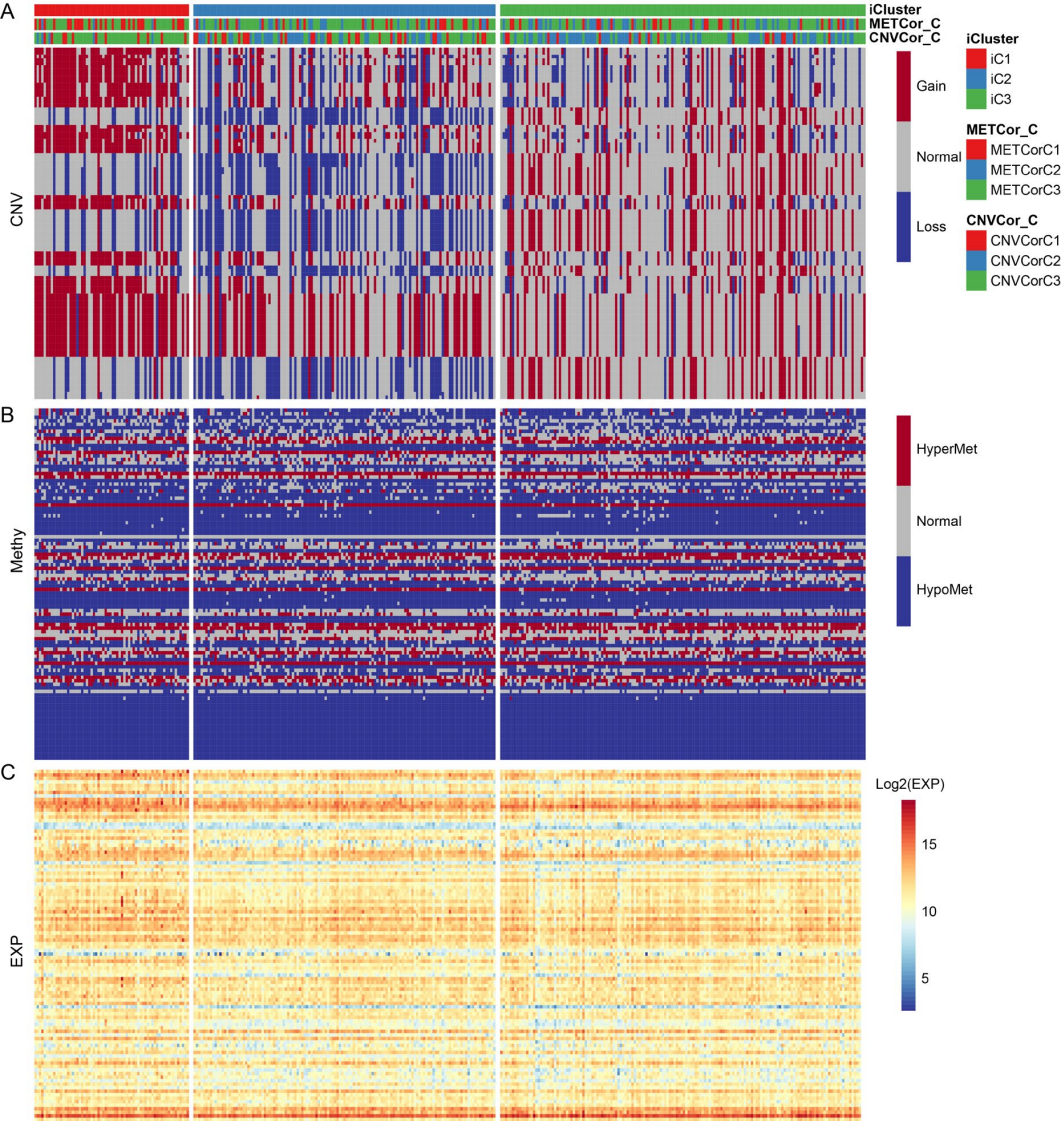
**The 100 genes with the most significant differences in CNV, Met, and gene expression among iC1 molecular subtypes.** (**A**) CNV distribution in iCluster gene subtypes; (**B**) MET distribution in iCluster gene subtypes; (**C**) Heatmap of differential genes for iCluster gene subtypes.

Differences in CNV, MET, and mRNA expression between the iCluster iC1 and iC3 subtypes, which also differed significantly in prognosis, were then analyzed (Figure S5). Samples were separated into the following three types based on CNV and MET data as previously described: CNV Gain (MetHyper), CNV Loss (MetHypo), and CNV Normal (MET Normal). Genes that differed significantly in CNV and MET data between the iC1 and iC3 subtypes were identified using the Fisher-exact test. Genes with differential expression based on gene expression (EXP) data between iC1 and iC3 subtypes were identified by DESeq2 (differential genes with p < 0.05). The genes that differed in CNV, MET, and expression spectrum are shown in Figure 6.

### Associations between gene expression, CNV, and MET

**Figure 7:**
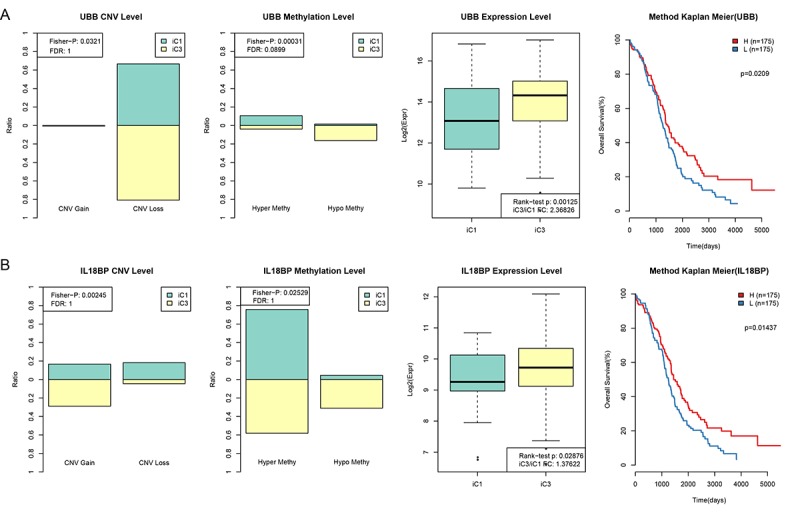
**CNV and MET in UBB and IL18BP were significantly correlated with prognosis.** (**A**) Comparison of CNV, MET, and EXP in the UBB gene between iC1 and iC3 subtypes and prognostic survival curve for the TIMM50 gene. From left to right: the proportion of UBB Gain and Loss (range: 0-1) in iC1 and iC3 samples, the proportion of UBB hypermethylation and hypomethylation (range: 0-1) in iC1 and iC3 samples, UBB expression in iC1 and iC3 samples, and survival curve with samples divided into high and low UBB groups based on median gene expression level. (**B**) Comparison of CNV, MET, and EXP in the IL18BP gene between iC1 and iC3 subtypes and prognostic survival curve for the TIMM50 gene. From left to right: the proportion of IL18BP Gain and Loss (range: 0-1) in iC1 and iC3 samples, the proportion of IL18BP hypermethylation and demethylation (range:0-1) in iC1 and iC3 samples, IL18BP expression in iC1 and iC3 samples, and survival curve with samples divided into high and low IL18BP groups based on median gene expression level. Survival time is shown on the x-axis and overall survival is shown on the y-axis; differences were identified using the log rank P test.

To investigate the relationship between gene expression, CNV, and MET, genes with both differential expression in the iC1 and iC3 subtypes and with a significant difference in CNV Gain/Loss and MetHypo/MetHyper (p < 0.05) were examined in a prognostic survival analysis. A total of 1,338 genes differing in CNV Gain/Loss, 11 genes differing in MetHyper/MetHypo, and 8,195 genes with differential expression between the iC1 and iC3 subtypes were included. Of the seven genes which differed in all three measures between the two subtypes (URI1, AKT2, ZHX3, RAB34, FBXO6, IL18BP, and UBB), two genes (UBB and IL18BP) were significantly associated with prognosis according to one-factor survival analysis (Figure 7. Table S8 and Table S9, p < 0.05). Specifically, low expression of both UBB and IL18BP was associated with poorer prognosis. Furthermore, in samples associated with poor prognosis, expression of these two genes was lower in iC1 subtype samples than in iC3 subtype samples, perhaps due to higher MET levels and lower CNV levels in the iC1 subtype.

### Survival analysis for UBB and IL18BP

**Figure 8:**
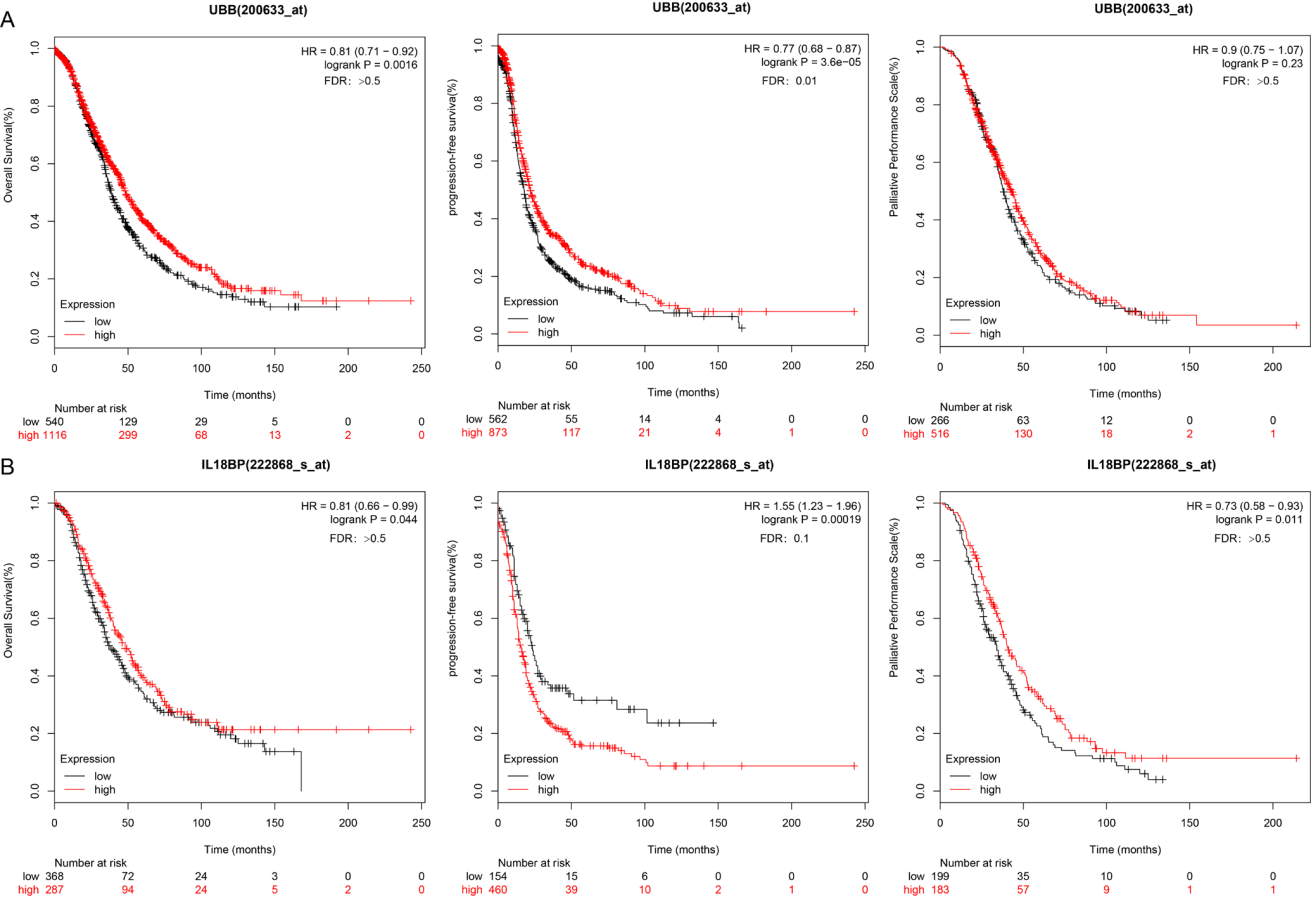
**UBB and IL18BP gene survival curves in the KMplot dataset.** (**A**) OS, PFS, and PPS curves for the UBB gene in a dataset from the KMplot database. Survival time is plotted on the x-axis; OS, PFS, and PPS are plotted on the y-axis. (**B**) OS, PFS, and PPS curves for the IL18BP gene in the KMplot dataset. Survival times are plotted on the x-axes; OS, PFS, and PPS are plotted on the y-axes. Differences were identified using the log rank P test.

Associations between the two genes of interest and prognosis were validated using 10 independent datasets from the KMplot website www.kmplot.com/lung. KM survival curves for OS, PFS (p < 0.05), and post-progression survival (PPS) for these genes are shown in Figure 8.

### Comparison of mutation spectrum in the OV gene subtypes

**Figure 9:**
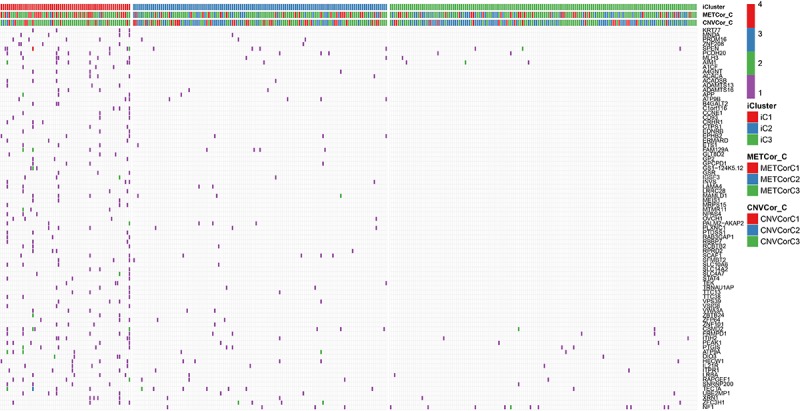
**Genes with differential mutations in the iC1 and iC3 molecular subtypes.** Different colors represent different numbers of mutations in the corresponding genes. iCluster indicates multi-omics molecular subtype, METcor_C indicates METcor gene molecular subtype, and CNVcor_C indicates CNVcor gene molecular subtype.

Based on the iCluster gene clustering results, we analyzed mutation spectra in the different subtypes and screened a set of genes that differed significantly between the iC1 and iC3 subtypes (Figure 9). In total, 83 genes with p < 0.05 were selected based on ranked Fisher test p values; associated data are shown in Table S10, and Fisher test results for SNV loci by group are presented in Table S11.

To investigate mutations in and expression of the UBB and IL18BP genes, we calculated the correlation between expression of these two genes at each SNV locus; the top ten loci are listed in Tables 3 and 4, and all results are shown in Tables S12 and S13. It was inferred from these results that the mutation loci significantly related to expression levels might have specific effects on UBB and IL18BP expression.

**Table 3:** Ten most significant correlations between UBB gene expression and SNVs.

gName	snvGene	SNV	CorrP	Corr
UBB	KMT2D	c.10830G>T	1.47E-05	0.286314
UBB	JMJD1C	c.5709G>A	1.47E-05	0.286314
UBB	FBXO24	c.950C>G	1.47E-05	0.286314
UBB	PTPN12	c.950A>T	1.47E-05	0.286314
UBB	PTPRN	c.905delG	1.47E-05	0.286314
UBB	TP53	c.844C>T	0.000834	0.222683
UBB	EDEM3	c.2548G>A	0.00124	0.215423
UBB	ARL11	c.88T>C	0.00124	0.215423
UBB	ZC3H12C	c.1649delC	0.00124	0.215423
UBB	LRRN3	c.1729delG	0.00124	0.215423

**Table 4:** Ten most significant correlations between IL18BP gene expression and SNVs.

gName	snvGene	SNV	CorrP	Corr
IL18BP	KLHL32	c.685_686insTTCCTGACTGTTAC	4.45E-07	0.331096
IL18BP	ARHGEF38	c.*1973_*1974insCTCTGGT	4.45E-07	0.331096
IL18BP	MMP21	c.890C>T	4.45E-07	0.331096
IL18BP	HSPA8	c.543_544insCCAAAACCATTCGTAGTTTCCACCAGAAA	4.45E-07	0.331096
IL18BP	ASXL3	c.4739_4740insACACCCGACCG	4.45E-07	0.331096
IL18BP	ACSF2	c.1519_1520insACA	4.45E-07	0.331096
IL18BP	PCDHA11	c.1774A>G	4.45E-07	0.331096
IL18BP	CCNB3	c.1083C>T	4.45E-07	0.331096
IL18BP	DUS1L	c.1327_1328insCAG	4.45E-07	0.331096
IL18BP	KRTAP9-9	c.202_203insG	4.45E-07	0.331096

## DISCUSSION

Several recent studies have shown that genomics, epigenomics, and transcriptomics play vital roles in tumor development and progression and can help predict patient prognosis [20]. Human tumor databases provide access to clinical and biological data from many large-scale studies using high-throughput sequencing technology and genomic chip technology. These databases thus serve as valuable resources for multi-omics analyses [21] which in turn enables identification of distinct molecular subtypes. Multi-omics investigations can therefore help identify new mechanisms underlying and clinically relevant definitions for tumor heterogeneity, candidate treatment targets, and tumor biomarkers [22].

In this study, a CNVcor gene set and a METcor gene set were identified using expression spectrum data from 351 ovarian carcinoma patients in the TCGA database. These 351 samples were subdivided into three subtypes (iC1, iC2, and iC3). Survival analysis showed that OS was much lower in the iC1 subtype than in the other two subtypes (iC2 and iC3). In addition, correlation analysis indicated that individuals presenting with DNA CNV might subsequently develop DNA MET variations. Such findings highlight the clinical need for multi-omics analysis of CNV data and MET data for early diagnosis and accurate prognosis predictions in ovarian carcinoma. We further characterized immune cell populations the three ovarian carcinoma subtypes. B cell scores were lower, while macrophage cell scores were higher, in iC1 subtype samples with the poorest prognoses compared to the other subtypes.

Differences in CNV, MET, and gene expression levels between the iC1 and iC3 subtypes were also examined. Combining gene expression with differences in CNV Gain/Loss and MetHyper/MetHypo allowed the identification of seven candidate genes and resulted in the largest difference in prognosis. The expression of two candidate genes, UBB and IL-18BP, was associated with OS; this finding was also validated using the GEO dataset. The results suggest that lower UBB and IL-18BP expression may be associated with higher MET and lower CNV levels; evaluating the expression of these genes might therefore aid in early tumor diagnosis and prognosis.

Finally, correlation analysis indicated that SNV gene mutation loci are significantly associated with UBB and IL18BP gene expression. In addition, mutation spectrum comparisons showed that overall mutation levels were higher in iC1 subtype samples than in iC2 and iC3 subtype samples, which might contribute to the poorer prognoses associated with the iC1 subtype.

The ubiquitin-encoding gene UBB is involved in several cancers, and suppression of UBB transcription plays a role specifically in ovarian carcinoma-specific changes [23]. Alexia *et al*. found that UBB expression was inhibited in approximately 30% of ovarian carcinoma patients, suggesting that UBB might be a promising treatment target [24]. In addition, recent studies indicate that the ubiquitin-proteasome system (UPS), which regulates many intracellular signaling pathways by controlling the expression, activity, and localization of various endogenous proteins, might be a promising target for cancer treatment in general [25]. Currently, a small number of UPS-targeting drugs (e.g., bortezomib) are available. These drugs, which are selective proteasome inhibitors, are very effective in treating refractory melanoma and mantle cell lymphoma [26]. These findings together with our results indicate that UBB might both serve as a new therapeutic target and assist in the diagnosis of and prognosis predictions in ovarian carcinoma.

The IL-18 is thought to bind to the protein products of IL18R1 and IL18RAP genes and has a high affinity with IL-18 binding protein (IL-18BP) [27]. Both *in vitro* and immunohistochemical experiments have shown that IL18BP can suppress the activity of endogenous or exogenous IL18 and interrupt its biological functions [28]. In addition, interactions between IL18, which is an immunity-enhancing cytokine, and IL18BP at the cell surface result in anti-tumor effects, including stimulation of T cell proliferation and increases in natural killer cell activity [29].

In our study, ovarian carcinoma patients with low IL18BP expression had poorer prognoses. In the tumor lesion microenvironment, increased expression of immunosuppressive molecules indicates a strong immune attack, which is beneficial for patients. Conversely, low levels of immunosuppressive molecules often suggest that the immune system is failing to recognize tumor lesions or is otherwise considerably damaged, ultimately resulting in a poor prognosis.

In conclusion, in this study we investigated possible pathogenic mechanisms of ovarian carcinoma via multi-omics data analysis of genomics, epigenomics, and transcriptomics. We found that DNA CNV and MET variation play important roles in ovarian carcinoma. In addition, we identified three potentially clinically relevant molecular subtypes of ovarian carcinoma and screened two key biomarkers. These novel mechanisms and clinical classifications might assist in the development of accurate diagnostic tests and treatments for ovarian carcinoma patients.

## MATERIALS AND METHODS

### Download of TCGA data

The most recent clinical follow-up data were obtained from the TCGA Genetic Disease Control (GDC) API on January 24, 2019; CNV, MET, and RNA-seq (including read count) data were also obtained for subsequent analysis of differential gene expression in different patient subsets. In addition, SNV data (mutect version) were downloaded from TCGA. Data from 351 patients in three datasets were included in the analysis; sample information for all three datasets is shown in Table S1.

### Profiling of DNA copy numbers, DNA methylation, mRNA expression, and SNV data

The CNV data were pre-processed as follows. Two regions with 50% overlap were considered identical. Regions covering <5 probes were deleted. The CNV region was mapped to corresponding genes using the GRCh38 release 22 (https://www.gencodegenes.org/human/release_22.html). Multiple CNV regions in a gene were merged into a single region, and CNV values were averaged to provide a merged CNV value. MET data were pre-processed by deleing absent loci in >70% of samples. Missing data were imputed using the KNN (k-Nearest Neighbor) algorithm. Probes in the TSS region from 2kb upstream to 200bp downstream were preserved using GRCh38 release 22 and mapped to the corresponding genes. RNA-seq data were pre-processed by deleting genes with low expression levels (FPKM = 0 in <0.5% of all samples). SNV data were pre-processed by deleting mutations in intron regions and silent mutations.

### Identification of CNVcor and METcor gene sets

The Pearson correlation coefficients for associations between CNV and RNA-seq and between MET and RNA-seq were calculated separately and converted into z-values using the formula ln((1+r)/(1-r)). Genes with p < 0.05 in the correlation coefficient test were included in the CNVcor and METcor gene sets. CNVcor and METcor gene data are shown in Table S2 and S3, respectively.

### Sample clustering via integration of CNV, MET, and gene expression data (EXP) data

The “iCluster” R package was used to conduct multi-omics clustering analysis by integrating CNV data from CNVcor genes, MET data from METcor genes, and EXP data from both CNVcor and METcor genes. Optimal weights for CNV, MET, and EXP datasets were determined based on lambda values. After completing 20 iterations to optimize lambda values, a total of 101 lambda sample points valued 0-1 were selected.

### Survival analysis

The KMplot website (http://kmplot.com/analysis/) was used to validate the data [30]. This database system contains integrated data from 8 independent datasets consisting of a total of 1,657 TCGA Ovarian Cancer (TCGA-OV) samples. Ovarian cancer patients were divided into 2 groups (high and low expression) based on the expression of the gene of interest. Kaplan-Meier plots were used to analyze overall survival in ovarian cancer patients. Hazard ratios (HR), 95% confidence intervals (CIs), and log rank P-values were evaluated.

## SUPPLEMENTARY MATERIAL

Supplementary Tables S1-S13

Supplementary Figures
