# Supplementary material for: Integrated multi-omics analysis of genomics, epigenomics, and transcriptomics in ovarian carcinoma
Source: Aging (Albany NY). 2019 Jun 29;11(12):4198–215. doi: 10.18632/aging.102047 (PMC6629004; doi:10.18632/aging.102047)
Supplement: Supplementary Figures [file aging-11-102047-s001.pdf]

SUPPLEMENTARY MATERIAL

Please browse the links in Full Text version of this manuscript to see Supplementary Tables.

Supplementary Tables S1 - S13.

Supplementary Figures

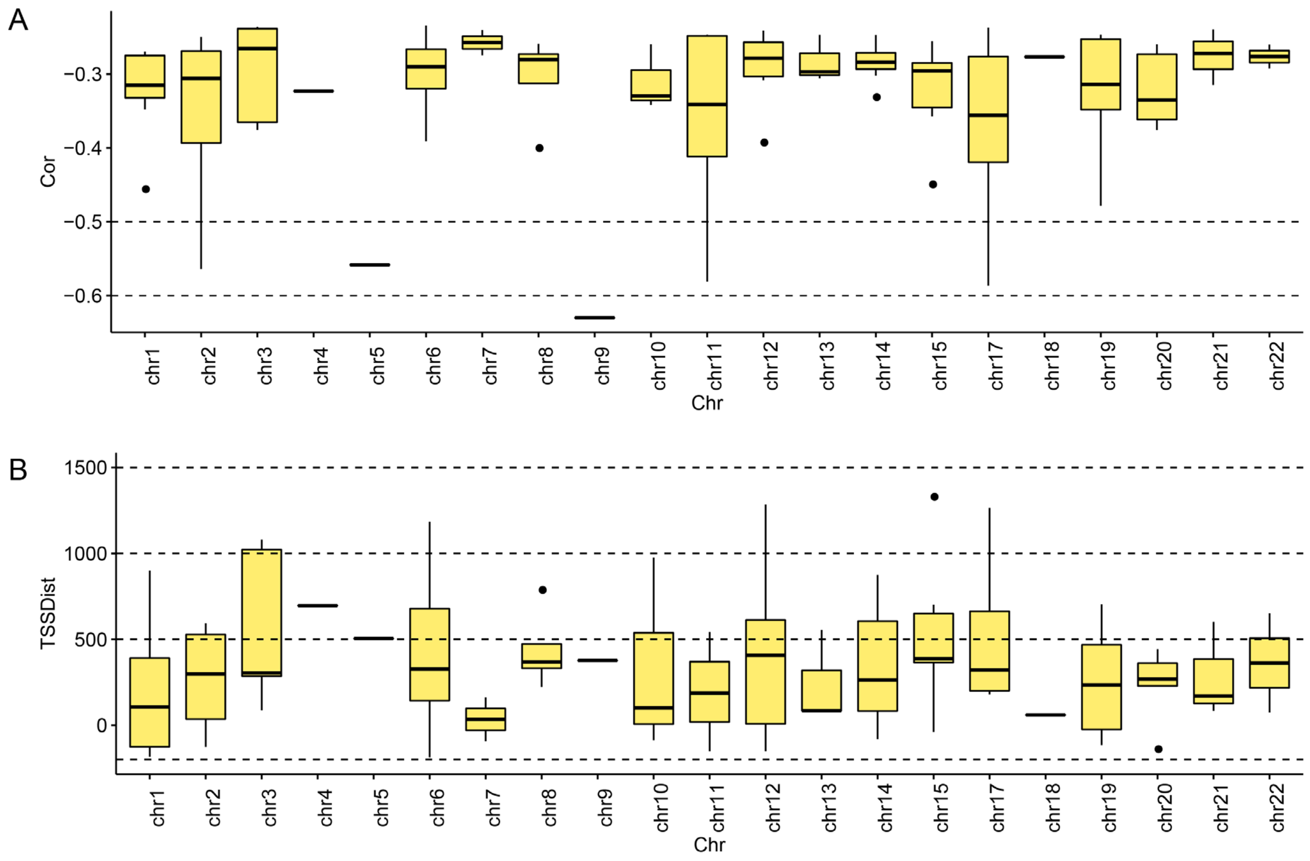

Figure S1. Box plot of METcor gene chromosome distribution and correlations. (**A**) Box plot of METcor gene correlation coefficients. The correlation coefficient of METcor genes on each chromosome is shown on the y-axis (**B**) Box-plot of METcor gene chromosome distribution. METcor methylated loci and the transcription start site (TSS) are shown on the y-axis.

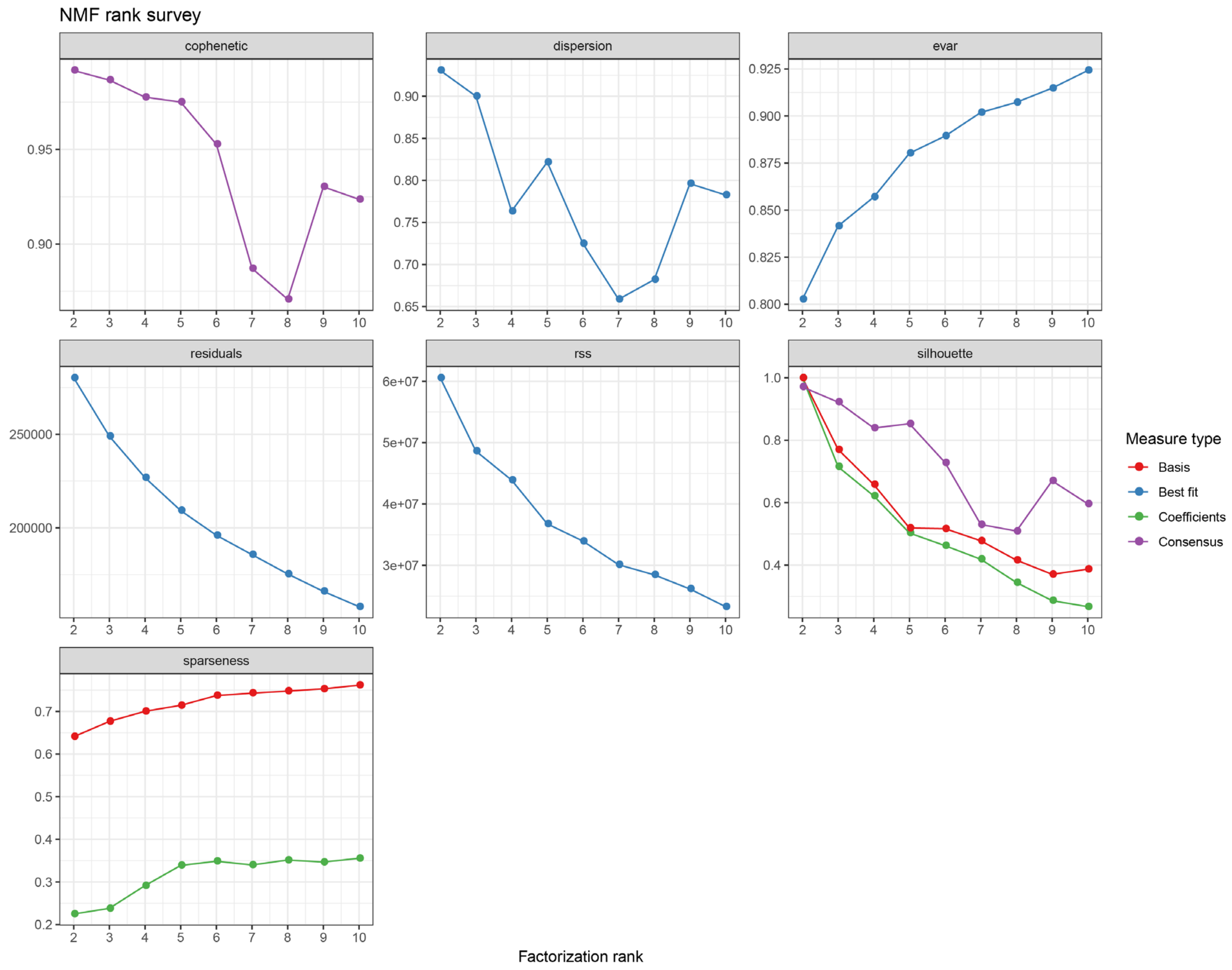

Figure S2. NMF clustering analysis for CNVcor genes. The NMF clustering method was used to evaluate the clustering effect from K=2-10. Cophenetic, dispersion, evar, residuals, rss, silhouette, and sparseness values were evaluated. The optimal clustering quantity was selected by combining these values in a consensus matrix.

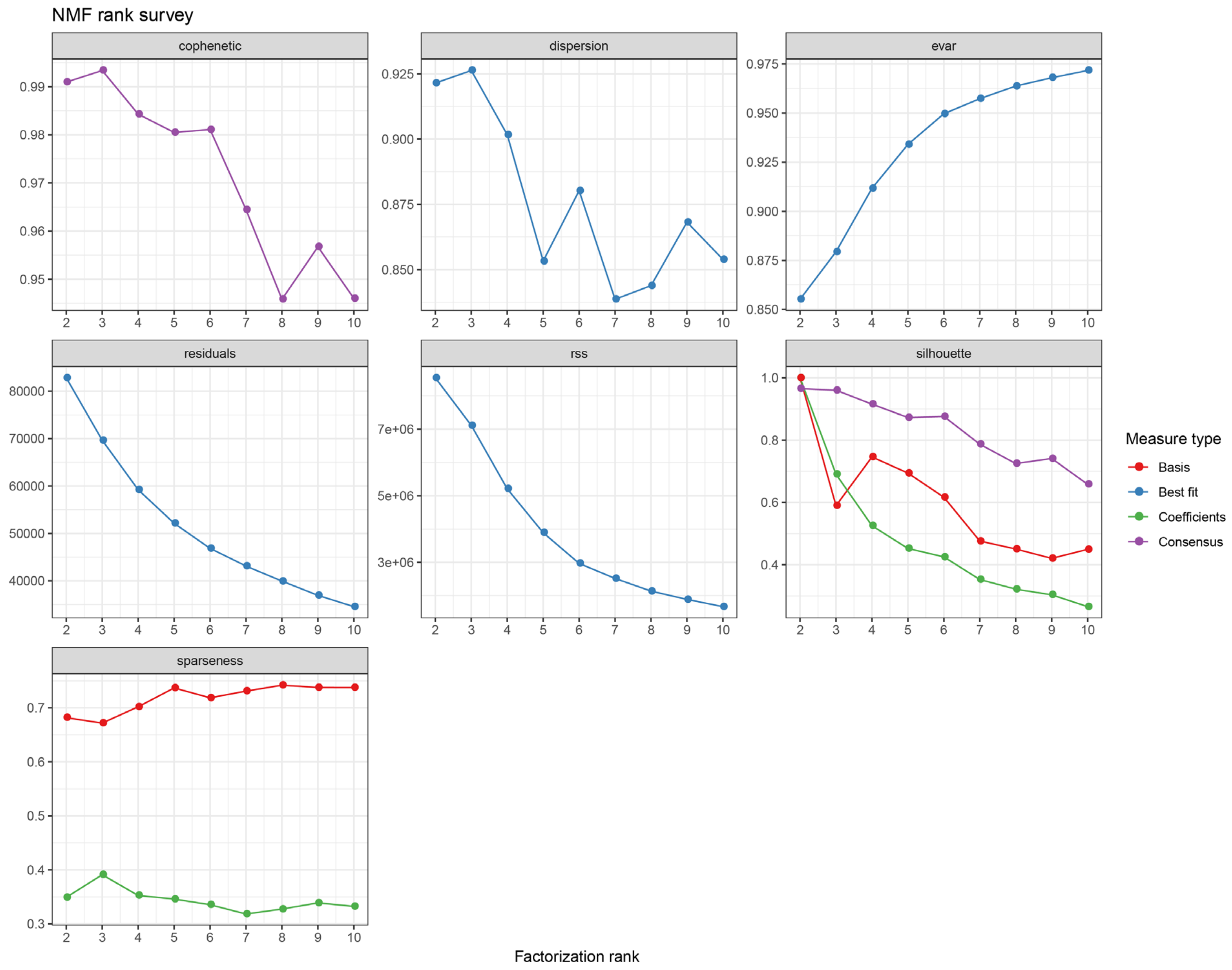

Figure S3. NMF clustering analysis of METcor genes. The NMF clustering method was used to evaluate the clustering effect from K=2-10. Cophenetic, dispersion, evar, residuals, rss, silhouette, and sparseness values were evaluated. The optimal clustering quantity was selected by combining these values in a consensus matrix.

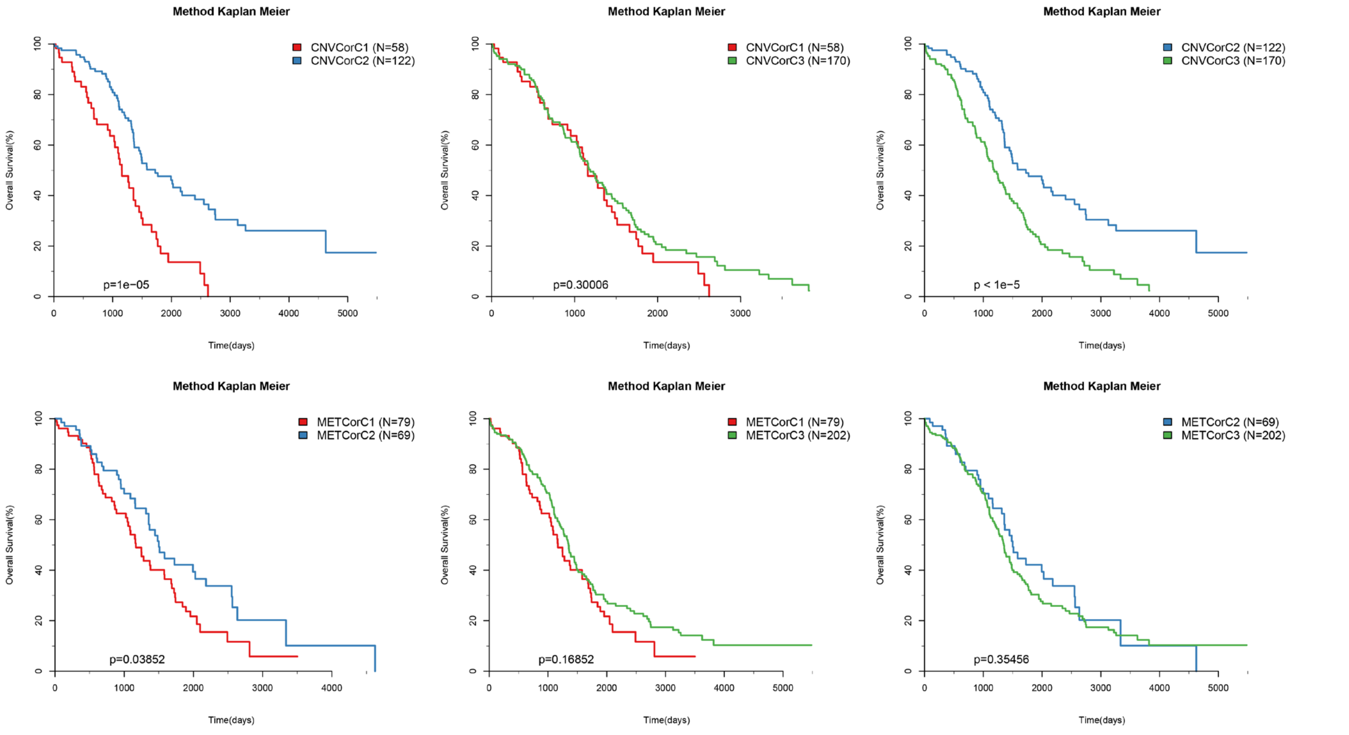

Figure S4. Differences in overall survival for both the CNVcor and METcor genes in the three subsets. KM survival curve for CNVcor gene or METcor gene clustering subsets. Survival time is shown on the x-axis, and survival rate determined by log rank P test is shown on the y-axis.

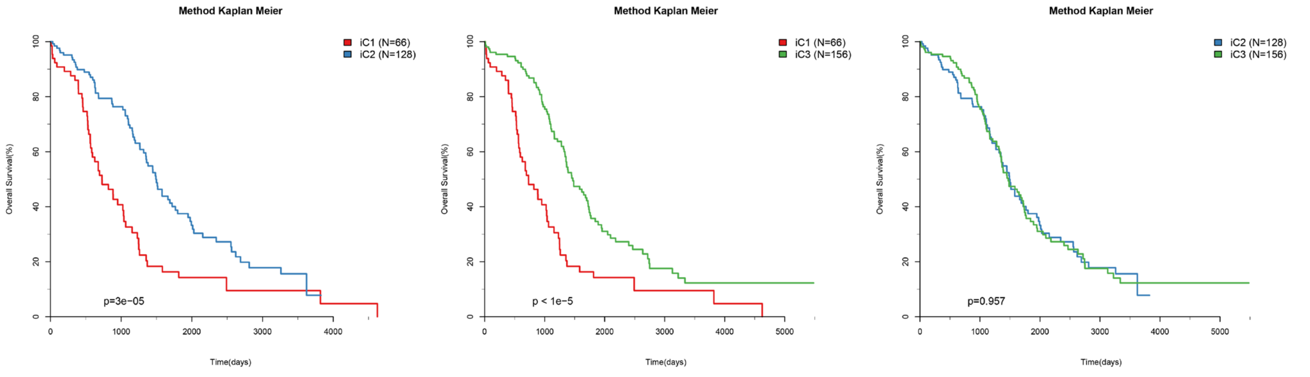

Figure S5. Prognostic differences among different molecular subtypes. The red line represents the iC1, blue line, the iC2, green line represents the iC3.Survival time is shown on the x-axis, and survival rate determined by log rank P test is shown on the y-axis.
